# IQ in Young Adulthood and Depressive Symptoms Over the Retirement Transition

**DOI:** 10.1093/geroni/igab046.2128

**Published:** 2021-12-17

**Authors:** Linn Elena Zulka, Valgeir Thorvaldsson, Linda B Hassing

**Affiliations:** University of Gothenburg, Gothenburg, Vastra Gotaland, Sweden

## Abstract

Retirement can be a challenging life transition for mental health. Higher levels of IQ in young adulthood have been shown to be advantageous for different outcomes later in life such as quality of life and well-being. However, it remains unclear whether possessing higher cognitive abilities in early life also favors individuals’ mental health when facing challenges related to the retirement transition. In this study, we therefore investigated the relationship between IQ in young adulthood and depressive symptoms over the retirement transition. We used data of six waves from the longitudinal population-based HEalth, Aging and Retirement in Sweden (HEARTS) study, as well as data on IQ in young adulthood from conscription. In a piecewise structural equation model, we modelled trajectories of depressive symptoms (measured by the CES-D scale) before and after retirement and in relation to young adulthood IQ (n = 1722 men). Results indicated an average decrease in depressive symptoms over the retirement transition for this sample of men. Higher childhood IQ was associated with further reduction in post-retirement depressive symptoms while controlling for education, retirement age, and memory ability and cardiovascular health at baseline. Our findings support the conclusion that higher IQ in young adulthood may act as a protective factor for mental health in the retirement transition. Individuals with higher IQ in young adulthood may have acquired coping strategies throughout their life-course, which they can apply when handling challenges related to retiring.

